# CMV+ Serostatus Associates Negatively with CD4:CD8 Ratio Normalization in Controlled HIV-Infected Patients on cART

**DOI:** 10.1371/journal.pone.0165774

**Published:** 2016-11-08

**Authors:** Isabelle Poizot-Martin, Clotilde Allavena, Claudine Duvivier, Carla Eliana Cano, Francine Guillouet de Salvador, David Rey, Pierre Dellamonica, Lise Cuzin, Antoine Cheret, Bruno Hoen, for the Dat'AIDS Study Group

**Affiliations:** 1 Aix-Marseille University, APHM Hôpital Sainte-Marguerite, Immuno-Hematology Clinic, Marseille, France; 2 Inserm U912 (SESSTIM), Marseille, France; 3 CHU Hôtel Dieu, Service des maladies infectieuses, Nantes, France; 4 APHP- Hôpital Necker—Service de Maladies Infectieuses et Tropicales, Centre d’Infectiologie Necker-Pasteur, Université Paris Descartes- IHU Imagine Paris, Paris, France; 5 Institut Pasteur, Centre Médical—Centre d’Infectiologie Necker-Pasteur, Paris, France; 6 Université Paris Descartes, Sorbonne Paris Cité, EA7327, Paris, France; 7 CHU Archet 1, Service de Maladies Infectieuses et Tropicales, Nice, France; 8 Hôpitaux Universitaires Strasbourg, Center for HIV care, Strasbourg, France; 9 Infectious Diseases Department, CHU of Nice, University Nice Sophia-Antipolis, Nice, France; 10 INSERM, UMR 1027, Toulouse III University, CHU Toulouse, COREVIH Toulouse, Toulouse, F-31000, France; 11 Paris- Descartes University, Sorbonne Paris-Cité, EA 3620, France; 12 Virology Laboratory, Necker Enfants-Malades Hospital, Paris, France; 13 Université des Antilles, Faculté de Médecine Hyacinthe Bastaraud, EA 4537, Pointe-à-Pitre, France; 14 Centre Hospitalier Universitaire de Pointe-à-Pitre, Inserm CIC1424, Service de Maladies Infectieuses et Tropicales, Dermatologie, Médecine Interne, Pointe-à-Pitre, France; University of Toronto, CANADA

## Abstract

Cytomegalovirus (CMV) infection is common among HIV-infected patients but its repercussion on the course of CD4+ and CD8+ T cells after cART initiation remains elusive. The French Dat'AIDS cohort enrolled 5,688 patients on first-line cART, from which we selected patients who achieved HIV suppression for at least 12 months without modification of cART, and for whom CMV serostatus was available. Five hundred and three patients fulfilled the selection criteria (74% male, median age 43 yrs, 15.5% CDC stage C), of whom 444 (88.3%) were seropositive for CMV (CMV+). Multivariate analyses using mixed-linear models adjusted for the time from HIV suppression, sex, age, transmission risk group, duration of HIV follow-up, the interaction between time from HIV suppression and CMV+ serology, and the nadir CD4 count revealed a negative correlation between CMV+ and CD4:CD8 ratio (coeff. = -0.16; p = 0.001). This correlation was also observed among patients displaying optimal CD4 recovery (≥500 cells/mm3 at M12; coeff. = -0.24; p = 0.002). Hence, CMV+ serostatus antagonizes normalization of the CD4:CD8 ratio, although further analyses of the impact of co-morbidities that associate with CMV serostatus, like HCV infection, are needed to elucidate this antagonism formally. However, this might reflect a premature T cell senescence, thus advocating for a close monitoring of T cells in CMV co-infected patients. In addition, our results raise the question of the benefit of treatment for asymptomatic CMV co-infection in HIV-infected patients.

## Introduction

HIV infection affects CD4+ and CD8+ T cells homeostasis by a depletion of CD4+ T cells and an expansion of CD8+ T cells in most antiretroviral-naïve HIV-infected patients [1]. After initiating combined antiretroviral therapy (cART), most HIV1-infected patients achieve sustained control of plasma HIV viremia associated with an increase of CD4+ T cell count [2]. However, many of them fail to restore CD4+ T cell count ≥500/mm3 and/or CD4:CD8 ratio ≥1. This might be linked to the persistence of a high CD8+ T cell count in the peripheral blood as recently reported [3]. Expansion of the CD8+ T cell compartment has been attributed to a persistent immune activation and inflammation, triggered by residual HIV replication, microbial translocation and chronic viral infections including viral hepatitis and CMV-infection [4].

CMV infection is highly prevalent among HIV-infected patients and its role as a risk factor for severe non-AIDS-defining events/non-AIDS-related death has been highlighted recently [5]. Furthermore, recent studies have also showed an inverse correlation between CD4:CD8 ratio and the risk of morbidity and mortality [6].

The aim of this study was to investigate the impact of CMV serostatus on the course of CD4+ and CD8+ T cells and of CD4:CD8 ratio in HIV-infected patients receiving a first-line cART.

## Methods

### Design and patients

This is a retrospective, observational, longitudinal analysis from “The Dat’AIDS Cohort”, a multicenter cohort of 26,959 HIV-infected patients from fifteen major French HIV centers using the NADIS^®^ electronic medical record (Fedialis Medica, Marly le Roi, France) for HIV, HBV- or HCV-infected adults [7, 8]. Patient data are recorded during medical visits in a structured database, and data quality is controlled systematically during capture, annual assessments and ad hoc processes prior to analyses [7, 8].

Selected subjects were HIV1-infected patients initiating a first-line cART between Jan-2002 and Dec-2009, achieving an undetectable plasma HIV load (pVL) for at least 12 months, and with available CMV serostatus (anti-CMV IgG positive or negative). Only patients with unchanged cART regimen through the study period were analyzed. CMV serology (CMV IgG) was determined by using EVOlyser (Tecan) with Behring’s reagents, and negative CMV serologies were controlled every year as per current French guidelines. All CMV seronegative patients were documented to still be seronegative at the end of the study period. The characteristics of patients were described at the time of initiation of first-line cART. T cell immunophenotyping (CD4+, CD8+ T cells and CD4:CD8 ratio) was performed from EDTA-fresh blood samples before cART initiation (pre-cART), at the first undetectable HIV-pVL (D0), and every 6 months during 3 years. According to the laboratory threshold values, normal counts were defined as 500–1200 cells/mm3 for CD4+ T cells, and 300–830 cells/mm3 for CD8+ T cells. Sex, age, duration of HIV exposure, transmission risk group, CDC stage, hepatitis C co-infection (HCV-RNA+), nadir CD4+ T cell count, HIV-pVL, and cART regimen (nucleotide reverse transcriptase inhibitor (NRTI)-, non-nucleoside/NRTI (NNRTI)-, or protease inhibitor (PI)-based) were analyzed.

Plasma HIV-RNA was quantified by successive standardized assays including Roche Cobas HIV-1 monitor, Roche Cobas Ampliprep/Cobas Taqman HIV-1v.2 test, and Abbott RealTime HIV-1 test with detection limits of 400, 200, 50 and 40 copies, respectively, according to the study period.

### Statistical Analysis

First, we compared patients’ characteristics at the time of ART initiation according to CMV serology using Student t-test (for continuous variables) or Pearson Chi-square tests (for dichotomous variables) as required (Table 1).

**Table 1:** Patients’ characteristics at the time of cART initiation.

	N (%)/ median [IQR]		
	CMV+ (n = 444)	CMV- (n = 59)	*p*^§^
Male	326 (73.4)	45(76.3)	ns
Age (years)	43[36 ; 51]	45[40 ; 49]	ns
Transmission risk group :			0.03
Heterosexual	15(3.4)	3(5.1)	
Homo/Bisexual	189(42.6)	15(25.4)	
Intravenous drug use	202(45.5)	33(55.9)	
Other	10(2.2)	4(6.8)	
Unknown	28(6.3)	4(6.8)	
HIV follow-up (yrs)	1[0.2 ;3.4]	3.4[0.3 ;8.4]	0.011
CDC staging:			ns
A	316(61.3)	37(62.7)	
B	62(14)	9(15.3)	
C	65(14.7)	13(22)	
HCV-RNA+	23(5.2)	3(5.2)	ns
CD4 Nadir (/mm3)	222[136 ;281]	221[157 ;282]	ns
First-line cART duration (months)	32[23.7 ;42.5]	32.6[23 ;43]	ns
Time to HIV suppression on cART (months)	3.05[1.9 ;5.2]	3.4[1.9 ;5.1]	ns
cART regimen :			ns
2 NRTI + 1 PI/r	223(52.2)	24(44.4)	
2 NRTI + 1 NNRTI	150(35.1)	23(42.6)	
3 NRTI	54(12.1)	7(13)	
Pre-cART HIV-pVL (Log_10_ copies/ml)	4.75[4.27 ;5.26]	4.67[4.26 ;5.05]	ns
Pre-cART CD4 (/mm3)	250[160 ;319]	261[165 ;321]	ns
Pre-cART CD4 (%)	16.7[11.1 ;22]	17.2[11.9 ;23.7]	ns
Pre-cART CD8 (/mm3)	777[537 ;1132]	755[491 ;1082]	ns
Pre-cART CD8 (%)	59.65[51 ;69]	57.2[47 ;63.8]	ns
Pre-cART CD4:CD8	0.3[0.2 ;0.4]	0.3[0.2 ;0.4]	ns
Pre-cART CD4 :CD8 ≥1	6(1.4)	2(3.4)	ns

^§^ p are Student t-test or Pearson Chi-square tests as required.

**Fig 1:**
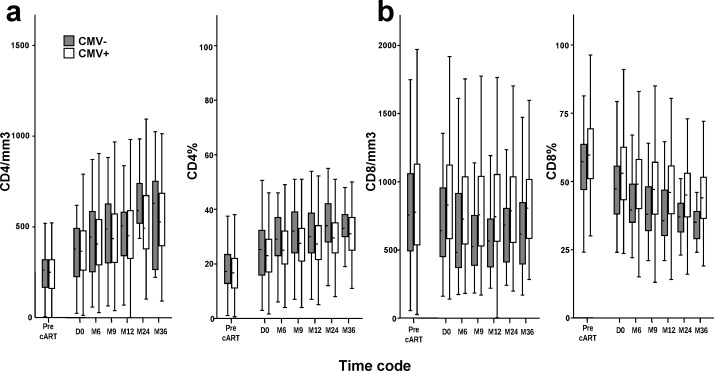
Longitudinal evolution of CD4+ and CD8+ T cell counts in CMV+ and CMV- HIV-infected patients on first-line cART. Box plots illustrate the median and interquartile ranges of CD4+ (a) and CD8+ (b) T cell counts and percentages in CMV+ (white) and CMV- (grey) HIV-infected patients on first-line cART.

Second, we analyzed the association between T cell dynamics and CMV serology using 5 different outcomes: CD4+ T cell count (cells/mm3), CD4%, CD8+ T cell count (cells/mm3), CD8% and CD4:CD8 ratio. We used mixed-linear model for repeated-measures to analyze the association between the 5 outcomes of T cell dynamics and CMV serology, time from D0 and the interaction between CMV serology and time from D0 (Fig 1 and Table 2).

**Table 2:** Mixed linear analysis of the dynamics of T cell compartments and CD4:CD8 ratio: association with CMV+ serology, time and interaction.

Variable	Estimation	(CI 95%)	*p*
CD4/mm3 :			
CMV+ serology	9.71	[-42.36 ; 61.79]	*0*.*712*
Time from D0 (months)	8.33	[6.20 ; 10.46]	*<0*.*001*
CMV+ serology * Time from D0	-0.94	[-3.21 ; 1.32]	*0*.*412*
CD4% :			
CMV+ serology	-2.24	[-4.91 ; 0.42]	*0*.*099*
Time from D0 (months)	0.34	[0.25 ; 0.44]	*<0*.*001*
CMV+ serology * Time from D0	-0.03	[-0.14 ; 0.07]	*0*.*492*
CD8/mm3 :			
CMV+ serology	195.70	[95.49 ; 295.92]	*<0*.*001*
Time from D0 (months)	-1.67	[-5.99 ; 2.64]	*0*.*447*
CMV+ serology * Time from D0	-2.13	[-6.70 ; 2.44]	*0*.*359*
CD8% :			
CMV+ serology	7.85	[4.23 ; 11.46]	*<0*.*001*
Time from D0 (months)	-0.46	[-0.68 ; -0.24]	*<0*.*001*
CMV+ serology * Time from D0	-0.01	[-0.25; 0.22]	*0*.*876*
CD4:CD8 ratio:			
CMV+ serology	-0.13	(-0.22 ; -0.04)	*0*.*006*
Time from D0 (months)	0.019	(0.015 ; 0.02)	*<0*.*001*
CMV+ serology * Time from D0	-0.006	(-0.010 ; -0.002)	*0*.*008*

**Fig 2:**
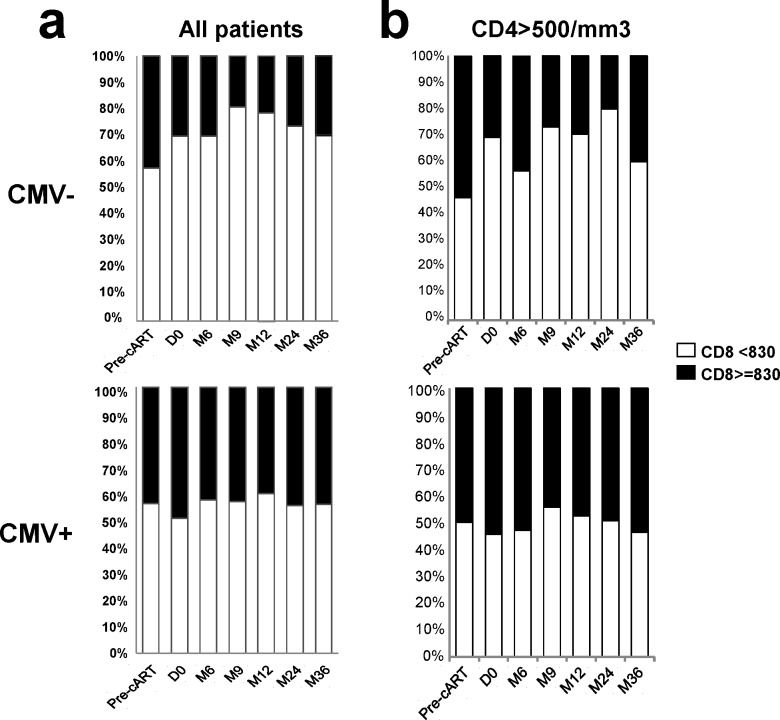
Longitudinal analyses of CD8+ T cell counts in CMV- and CMV+ HIV-infected patients on first-line cART. 100% stacked bar charts illustrate the fraction of patients displaying CD8+ T cell counts ≥830/mm3 (black) and <830/mm3 (white) among all study participants (a) or in patients achieving CD4+ T cell count ≥500/mm3 after 12 months of HIV suppression on cART (b) in CMV- (top) and CMV+ (bottom) patients.

Third, we analyzed the evolution of T cell compartments: CD4+ T cell counts were dichotomized according to the threshold of ≥500 cells/mm3; CD8+ T cell counts using the threshold of ≥830 cells/mm3 (Table 3, Fig 2). We used mixed models with logit distribution for binary variables entering CMV serology and the time from D0 as covariates (data not shown). The interaction term was tested but not included in these models as it was never significant. We computed a sub-analysis of CD8+ T cell compartments in patients achieving CD4+ cell count ≥500/mm3 after 12 months of HIV suppression on cART (Fig 2B).

**Table 3:** Recovery of the CD4+ T cell compartment in CMV seropositive and CMV seronegative HIV-infected patients on first-line cART.

		N(%)				
CD4/mm3		Pre-cART	Day 0*	M12	M24	M36
**≥ 500**	CMV+	11(2.5)	93(21.3)	164(40.5)	92(48.2)	47(56)
	CMV-	2(3.4)	13(22)	28(52.8)	14(73.7)	7(70)
**<500**	CMV+	433(97.5)	344(78.7)	241(59.5)	99(51.8)	37(44)
	CMV-	57(96.6)	46(78)	25(47.2)	5(26.3)	3(30)

*day 0 is the time of first undetectable HIV pVL.

**Fig 3:**
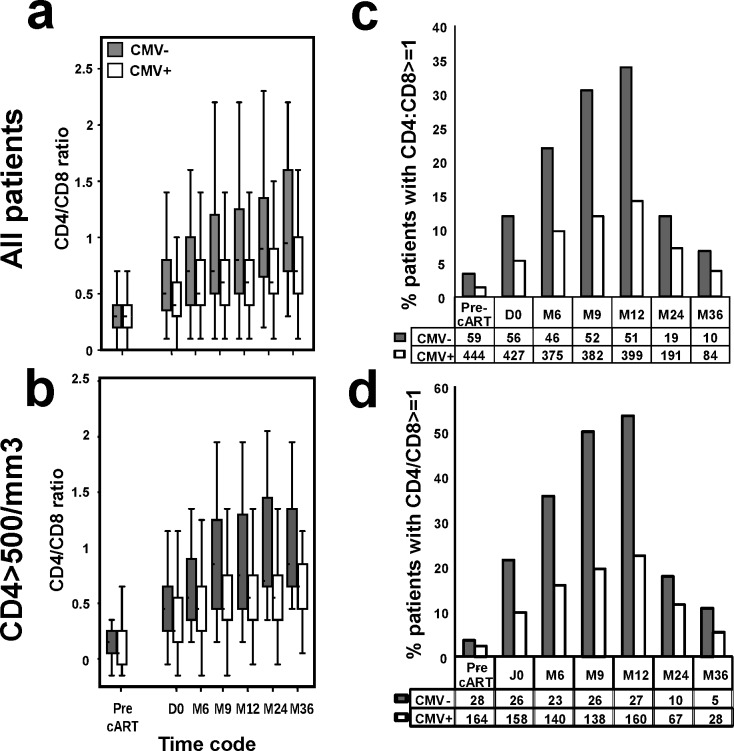
Longitudinal analyses of CD4:CD8 ratio in CMV+ and CMV- HIV-infected patients on first-line cART. Data are shown for all study participants (a and c) or patients achieving CD4+ cell counts ≥500/mm3 after 12 months of HIV suppression on cART (b and d). In a and b, box plots illustrate median (IQR) CD4:CD8 ratio, and in c and d, histograms show the percentage of patients presenting a CD4:CD8 ratio ≥1. All data are shown prior to cART initiation (Pre-cART), at first undetectable HIV-pVL (D0) and at 6, 9, 12, 24 and 36 months from viral suppression.

We then analyzed the CD4:CD8 ratio using a linear mixed model (Table 2) and, when looking at the percentage of patients with CD4:CD8 ratio ≥1, we used a logit mixed model entering CMV serology, the time from D0 and the interaction term for both models. We also computed a sub-analysis of the percentage of patients with a CD4:CD8 ratio ≥1 among patients achieving CD4+ cell count ≥500/mm3 after 12 months of HIV suppression on cART (Fig 3D).

We finally built multivariable models (Table 4) for the tree outcomes that were significantly associated with CMV serology in Table 2 (CD8/mm3, CD8%, CD4:CD8 ratio). Final multivariable models included correlates who had a p-value <0.20 in the univariable models (data not shown). Correlates tested in univariable models were 1) socio-demographic characteristics (sex and age); 2) HIV-related characteristics (Transmission risk group, CDC staging, pre-cART HIV viral load (pVL), nadir CD4 count), CMV serology, time from D0 and the interaction between CMV serology and the time from D0. The interaction term between time and CMV+ serology was forced in the final multivariable models. Analyses used SPSS Advanced Statistics 20 (IBM Corp) and SAS version 9.4 for Windows.

**Table 4:** Multivariate linear-mixed model analyses of factors associated with CD8+ T cell counts, CD8% and CD4:CD8 ratio.

	CD8/mm3			CD8%			CD4 :CD8		
	Coef	IC	*p*	Coef	IC	*p*	Coef	IC	*p*
Time from D0 (months)	-3.05	[-4.05 ; -2.05]	<10^−3^	-0.38	[-0.42;- 0.33]	<0.001	0.017	[0.014 ; 0.019]	<10^−3^
Sex (female vs male)	-66.3	[-140.63 ; 8]	0.08	-3.49	[-6.54; -0.45]	0.025	0.13	[0.05 ; 0.2]	<10^−3^
Age	2.34	[-.68 ; 5.37]	0.13	0.18	[0.07 ; 0.28]	0.001	-0.002	[-0.004 ; 0]	0.10
Transmission risk group :									
Heterosexual		na		1			1		
Homo/Bisexual				-3.82	[-6.66 ; -0.98]	0.008	0.08	[0.01 ; 0.11]	0.03
Intravenous drug use				-4.16	[-10.82 ; 2.5]	0.220	0.037	[-0.12 ; 0.19]	0.64
Other / Unknown				-1.97	[-5.94 ; 2.01]	0.331	-0.033	[-0.12 ; 0.06]	0.50
HIV follow-up (yrs)	13.7	[6.3 ; 21.1]	<10^−3^	0.418	[0.15 ; 0.69]	0.003	-0.009	[-0.02;-0.002]	0.01
CDC staging :	na						na		
A				1					
B				-0.83	[-4.05 ; 2.38]	0.611			
C				2.62	[-0.78 ; 6.03]	0.130			
Pre-cART HIV-pVL (Log)		na		-0.36	[-1.1 ; 0.38]	0.336	na	na	na
Nadir CD4 count (/mm3)		na		-0.03	[-0.04 ; -0.02]	<0.001	-0.0093	[-0.015 ;-0.002]	0.01
CMV+ serology	220.2	[118.5 ; 321.9]	<10^−3^	9.72	[6.25 ; 13.18]	<0.001	-0.006	[-0.009 ; -0.004]	<10^−3^
CMV+ serology *Time from D0 (months)	-0.94	**[**-4,2 ; 2,3]	0.57	-0.018	[-0.16 ; 0.12]	0.8	-0.006	[-0.009 ; -0.004]	<10^−3^

### Ethics statement

All subjects participating to this study provided written informed consent for the use of their medical records on the NADIS database, which was approved by the French data protection agency “Commission Nationale Informatique et Liberté” (Registration number: 2001/762876/nadiscnil.doc). All patients’ records were anonymized and de-identified prior to the analysis. This study was carried out in compliance with the International guidelines for human research protection as per the Declaration of Helsinki and ICH-GCP.

## Results

### Patients’ characteristics according to CMV serology

Of 5,688 patients who initiated a first-line cART during the study period, 503 fulfilled the selection criteria, of which 210 maintained their first-line cART regimen until M24, and 94 until M36. Their median duration of HIV follow-up was 32 months (IQR: 23.7–46.2) and 88% had positive CMV serology. The study participants were distributed in two groups according to CMV serostatus, group CMV+ (n = 444) and group CMV- (n = 59). Patients’ characteristics at the time of cART initiation (Pre-cART) are shown in Table 1. Both groups were similar excepting for the shorter duration of HIV infection and the higher proportion of homosexual/bisexual HIV transmission in the CMV+ group. Pre-cART CD4+ and CD8+ T cell counts, as well as CD4:CD8 ratio, did not differ significantly according to CMV serology.

### T-cell dynamics after HIV suppression on first-line cART

#### CD4+ and CD8+ T cell counts and percentages

The kinetics of CD4+ and CD8+ T cell counts and percentages in CMV+ and CMV- patients are shown in Fig 1. The analyses of the evolution of T cell counts and percentages according to CMV serology by using a mixed-linear model (Table 2), showed that CD4+ T cell counts and CD4% increased significantly over time, but were not significantly different by CMV serostatus. In contrast, CD8+ T cell counts did not vary significantly over time, while CD8% decreased significantly. Both CD8+ T cell counts and CD8% were associated with CMV+ serology.

#### Recovery of the CD4+ T cell compartment

In addition, we observed a smaller proportion of patients achieving CD4+ T cell recovery ≥500 cells/mm3 in the CMV+ group compared to the CMV- group (Table 3), although this difference failed to achieve significance in a mixed model analysis using logit distribution (data not shown, p = 0.09). In contrast, CD4 recovery (≥500 cells/mm3) was significantly associated with the time from D0 (p<0.0001).

#### Dynamics of the CD8+ T cell compartment

Classification of patients by ranges of CD8+ T cell counts (<830 and ≥830 cells/mm3) revealed differences between CMV+ and CMV- patients. As shown in Fig 2, starting from D0, we observed a higher proportion of CMV+ patients displaying CD8+ T cell counts ≥830 cells/mm3 compared to CMV- patients (Fig 2A). This was still observed when focusing on patients who presented optimal CD4 recovery (CD4+ T cell counts ≥500/mm3 after 12 months of HIV suppression; Fig 2B). The distribution of patients at the time of cART initiation according to CD8+ T cell counts (<830; ≥830 cells/mm3) was similar between CMV- and CMV+ patients. Mixed model analysis using logit distribution revealed that CMV+ serology was associated with CD8+ T cell counts ≥830 cells/mm3 (p = 0.003), even in patients with CD4 ≥500 cells/mm3 (p = 0.02; data not shown). The time from D0 was negatively associated with CD8+ counts ≥830 cells/mm3 (p = 0.001). The interaction term between CMV+ serology and the time from D0 was not significantly associated with this outcome (data not shown).

#### CD4:CD8 ratio

As shown in Fig 3, CMV- and CMV+ patients had similar median CD4:CD8 ratio pre-cART, and similar proportion of patients with a CD4:CD8 ratio ≥1. However, after HIV suppression, CMV- patients displayed higher median CD4:CD8 ratio (Fig 3A and 3B) and higher percentage of patients with CD4:CD8 ratio ≥1 (Fig 3C and 3D). This was also true in patients achieving optimal CD4+ T cell recovery (Fig 3B and 3D).

Analysis using a mixed linear model showed that CD4:CD8 ratio increased significantly over time (Table 2, bottom), and the probability of having a low CD4:CD8 ratio is higher in CMV+ patients. The interaction between the time from D0 and CMV serostatus was significant, indicating that the increase of CD4:CD8 ratio over time is less important in CMV+ patients compared to CMV- ones.

A mixed logit model revealed that CMV+ serology was negatively associated with the proportion of patients with CD4:CD8 ratio ≥1 (p = 0.001), even among patients with CD4 ≥500 cells/mm3 (p = 0.04; data not shown). The time from D0 was associated positively with CD4:CD8 ratio ≥1 (p = 0.001). The interaction term between CMV+ serology and the time from D0 was not significantly associated with this outcome (data not shown).

### Multivariable final model

Results of multivariable analysis are presented in Table 4. For CD8+ T cell counts, potential confounding factors identified were the time from HIV suppression, sex, age and the duration of HIV follow-up. For CD8%, potential confounding factors were the time from HIV suppression, sex, age, transmission risk group, duration of HIV follow-up, HIV-pVL (Log_10_), CDC staging and nadir CD4 count. For CD4:CD8 ratio, the potential confounding factors identified were the time from HIV suppression, sex, age, transmission risk group, duration of HIV follow-up, the interaction between time from HIV suppression and CMV+ serology, and nadir CD4 count.

Multivariable mixed-linear analysis adjusting for the variables indicated above confirmed the significance of the association of CMV serostatus with CD8+ T cell count, CD8% and CD4:CD8 ratio. Moreover, in the CD4:CD8 ratio model, the interaction of CMV+ serology with the time from D0 was significant and confirmed that the CD4:CD8 ratio has a lower increase over time in CMV + patients compared to their CMV- counterparts.

Then, we focused on patients that achieved an optimal CD4+ T cell reconstitution (≥500 cells/mm3 at M12), and we applied the same multivariate models described above (data not shown). In this sub-population, the associations of CMV+ serology with CD8+ T cell outcomes and the CD4:CD8 ratio were confirmed (CD8/mm3: coeff. = 215; 95% CI[47.78; 382.22]; p = 0.012, CD8%: coeff. = 10.71 [5.94; 15.49]; p<0.001 and CD4:CD8 ratio: coeff. = -0.24 [-0.38; -0.09]; p = 0.002).

## Discussion

This study performed on HIV-infected patients with sustained control of HIV replication on first-line cART, showed that the restoration of T cell balance is less efficient in patients bearing a CMV+ serology. The observations made in this study indicate that CMV-coinfection may support the expansion of the CD8+ T cell compartment and counter the improvement of the CD4:CD8 ratio.

Interestingly, we observed that the association of CMV+ serology with the failure to normalize CD4:CD8 ratio was significant even in patients with an optimal CD4+ T cell recovery. Since a low CD4:CD8 ratio was associated with increased risk of morbidity and mortality in HIV-infected patients [6], high CD8+ T cell counts may have clinical relevance in HIV disease by impairing the normalization of CD4:CD8 ratio. Hence, it appears from our study that CMV+ serology is among the risk factors that correlate with a persistently low CD4:CD8 ratio, similar to T cell activation and immunosenescence in HIV-infected patients on cART.

Our results are consistent with other recent studies performed on smaller samples of HIV-infected adults and children [9, 10]. However, our study selected patients that achieved HIV suppression for at least twelve months without cART modification, which allowed to rule out the action of HIV replication on the course of T cell counts, unlike Barrett’s and Kapetanovic’s studies, in which viral suppression was not achieved in all study participants. Furthermore, since we selected patients receiving their first-line cART, we addressed the question of the impact of CMV serostatus on the initial response to antiretroviral treatment, which could not be investigated in these studies.

In the general population, the CD4:CD8 ratio gradually declines with age and is negatively correlated with the proportion of senescent CD8+ T lymphocytes. Hence, CD4:CD8 ratio is considered a surrogate marker for immunosenescence [11, 12]. Interestingly, the median age in our cohort was 43[36; 51] years-old in CMV+ patients and 45[40; 49] years-old in CMV- patients, whereas CD4:CD8 ratio was <1 in most patients at baseline (96% CMV- and 98% CMV+ patients). After 12 months of HIV suppression, 52.5% of CMV- vs. 76% of CMV+ patients presented with an inversion of CD4:CD8 ratio, suggesting that CMV+ patients may present with a higher level of premature immune senescence. This is consistent with prior observations in HIV-uninfected individuals [13] and with Barrett’s report of fewer CD28- senescent cells in CMV seronegative HIV-infected adults [9].

Previous studies reported a premature immune senescence in naive HIV-infected patients, characterized by an increased proportion of CD28- T cells, without consistent normalization on cART[14, 15]. Kapetanovic and colleagues [10] observed that in HIV-infected children experiencing virological failure on cART, those with CMV- serology displayed a faster decrease of the proportion of CD8+CD28- T cells upon cART modification, regardless of their virological response to treatment. Hence, it appears that CMV coinfection supports the resilience of T cell senescence to antiretroviral treatment.

In the elderly population, the concomitant presence of an inverted CD4:CD8 ratio, CMV+ serology, an increased proportion of senescent CD8+CD28- lymphocytes, and poor T cell proliferative responses defines the immune risk phenotype (IRP)[12]. The IRP has been associated with higher risk of mortality and morbidities, including vascular disease and osteoporosis [12, 16]. Consistently, a pilot study on HIV-infected patients found that IRP is accompanied by telomere shortening, weak proliferative responses and enhanced production of proinflammatory cytokines[17]. An association between chronic CMV infection, immune senescence and activation was previously reported in HIV-infected patients [18], and CMV coinfection was recently identified as a risk factor for severe non-AIDS-defining events/non-AIDS-related death, cerebrovascular and cardiovascular events [5]. In addition, CD4:CD8 ratio is strongly correlated with activation, exhaustion and expression of senescence markers in naive HIV-infected patients [19], and in patients with long term HIV suppression on ART [20].

Unfortunately, the retrospective design of our study has several limits. Our study did not allow evaluating immune activation, inflammation or measuring indicators of bacterial translocation, the proportion of CD8+CD28- cells or telomere length. Prospective studies should be performed in order to determine the role played by these factors through quantitative assessments. Furthermore, we did not collect information on comorbidities or AIDS/non-AIDS-clinical events. Therefore, we could not explore the impact of CMV coinfection on the correlation between CD4:CD8 ratio and such events. Moreover, we cannot exclude that socio-economic factors (including nutrition, precarious accommodation or other sanitary issues) that were not assessed in our study, might have an impact on the immune response to cART. However, the fact that all study participants had sustained undetectable HIV viral load during the study period indicates high levels of adherence to cART.

In conclusion, in the context of an efficient HIV suppression after cART initiation, CMV+ serostatus is associated with a resilient expansion of the CD8+ T cell compartment and with an inverted CD4:CD8 ratio. This might reflect a premature T cell senescence in CMV+ patients. So far, data from the general population suggests that immune senescence is not reversible [12], and data on HIV infected patients is scarce on this matter. Valganciclovir treatment has proved efficient to reduce the proportion of activated CD8+CD38+HLA-DR+ T cells in HIV-infected patients [21]. Based on these observations and ours, it can be hypothesized that CMV-targeted adjuvant therapy might be beneficial for HIV-infected patients on cART by acting on the CD8 compartment. Furthermore, our results advocate for a close monitoring of both CD4+ and CD8+ T cells in HIV-infected patients with CMV+ serostatus. Finally, this study supports that CMV serological status should be taken into account when measuring the effectiveness of antiretroviral therapy on immune restoration, and might have an impact on the effectiveness of future cure approaches.

## Supporting Information

S1 Dataset Patients’ Characteristics and T cell values. (XLSX)
